# The financial losses from the migration of nurses from Malawi

**DOI:** 10.1186/1472-6955-5-9

**Published:** 2006-11-02

**Authors:** Adamson S Muula, Ben Panulo, Fresier C Maseko

**Affiliations:** 1 Department of Community Health, University of Malawi, College of Medicine, Blantyre, Malawi; 2 Department of Epidemiology, School of Public Health, University of North Carolina at Chapel Hill, North Carolina, USA; 3 Medical Student, University of Malawi, College of Medicine, Blantyre, Malawi; 4 Malawi College of Health Sciences, Lilongwe, Malawi

## Abstract

**Background:**

The migration of health professionals trained in Africa to developed nations has compromised health systems in the African region. The financial losses from the investment in training due to the migration from the developing nations are hardly known.

**Methods:**

The cost of training a health professional was estimated by including fees for primary, secondary and tertiary education. Accepted derivation of formula as used in economic analysis was used to estimate the lost investment.

**Results:**

The total cost of training an enrolled nurse-midwife from primary school through nurse-midwifery training in Malawi was estimated as US$ 9,329.53. For a degree nurse-midwife, the total cost was US$ 31,726.26. For each enrolled nurse-midwife that migrates out of Malawi, the country loses between US$ 71,081.76 and US$ 7.5 million at bank interest rates of 7% and 25% per annum for 30 years respectively. For a degree nurse-midwife, the lost investment ranges from US$ 241,508 to US$ 25.6 million at 7% and 25% interest rate per annum for 30 years respectively.

**Conclusion:**

Developing countries are losing significant amounts of money through lost investment of health care professionals who emigrate. There is need to quantify the amount of remittances that developing nations get in return from those who migrate.

## Background

The healthcare delivery systems of many countries in Africa are unable to deliver adequate quality and quantity of services attributable to, among other reasons, the shortage of health professionals. In Malawi, the high maternal mortality ratio estimated at about 1120 deaths/100,000 live births has been partly blamed on the unavailability of trained midwives to deliver satisfactory quality intra-partum care [1]. There have also been concerns that the rapid scaling-up of antiretroviral therapy aimed at serving about 170,000 Malawians will be constrained by non-availability of adequately trained nurses, clinical officers and doctors. Health care at district level facilities suffers the most from these human resources shortages as they are rural-based and have difficulties recruiting health workers. As a consequence, many cases requiring general care are referred to central hospitals [2]. The shortage of adequate health human resources in Malawi has been described as a 'crisis' by some authors [3].

The inadequate numbers and quality of health professionals in the health system arise from several factors such as inadequate output from the training institutions [4], poor motivation and the migration of health professionals to developed nations [5,6]. In the past few years, this issue of migration or 'brain drain' has attracted attention and has been described in the medical and health services literature. Various 'push', 'pull' and 'grab' factors have been described as fueling the losses of health professionals from Africa [7,8].

While many African countries have suffered the brain drain of their health human resources to various degrees, Malawi is among the countries worst affected by shortage of human resources. In absolute terms the numbers of health professionals who have out-migrated from Malawi may be surpassed by other countries, such as Kenya, South Africa and Zimbabwe, however as a proportion of the available health workforce, Malawi's losses are significant. For example, out of an estimated 4000 nurses in active service in Malawi in 2005, 453 nurses who had been trained in Malawi were reported to be working in OECD countries [9]. This represented 11.3% of the number of nurses active service in the country at the time.

In Malawi the entry requirements for enrollment into nurse-midwife training is the Malawi School Certificate of Education (Ordinary Level). Primary school is for 8 years, and secondary education is for 4 years, at which time the school certificate examinations are written. Enrolled nurse and midwifery training is for 3 years and is offered at the Malawi College of Health Sciences and any of the 8 mission nursing schools scattered across the country mostly in rural mission hospitals.

While the numbers of health professionals that have emigrated from sub-Saharan Africa, including Malawi are of interest, are increasingly being reported in the literature [10,11], the next step is to estimate the cost of training and expected lost investment from the migration. We therefore present a financial analysis and estimate of the financial loss Malawi experiences because of out-migration of nurses trained at two levels. This could contribute to policy debates and health services research on the economic impact of brain drain on the African continent.

## Methods

The cost of primary school was determined by obtaining the fees paid by students a private school (Kaphuka Primary School) and a mission school (Malamulo SDA School). The cost of secondary education was obtained from secondary schools associated with these primary schools. These schools are self-sufficient.

The cost of professional or tertiary education was obtained through interviews with relevant administrators of the training institutions and review of records. The administrators were asked the amount of money that the institutions spent per student per year. For the Kamuzu College of Nursing (KCN) especially, reported annual per capita expenditure was compared to cost estimates reported by Namate [4]. It was found that the estimated costs were not much different.

The health professional categories/cadres chosen for this study were those that normally migrate out of Malawi. Other health professional cadres such as pharmacy and laboratory staff may migrate but are considered to be in short supply mainly because of low production output.

One way to estimate the total cost of educating a health professional, is total the tuition fees at self-sustaining at primary and secondary school and then add cost for the health professionals' training tuition and other fees (e.g. nursing school).

### Loss of investment

Estimation of per capita loss was determined by calculating a future value (FV) of investment for a fixed sum of money at a particular interest rate. These methods are similar to those used by Kirigia et al [12] and Kennedy [13]. The FV i.e. loss from a country through migration was estimated as: FV = Sum * (1+r)^n ^where; Sum = amount invested; r = compound interest rate; n = the number of years the money is invested.

### Interest rates at banks

For the purposes of this study, calculation of capital losses was based on the prevailing fixed deposits and mortgage commercial bank rates (r)in Malawi in May 2006. The rates were as follows: National Bank, fixed deposit of 7% pa and mortgage of 25%, NBS Bank fixed deposit 10% and 25% mortgage, Opportunity International Bank, fixed deposit 10% and no mortgage product and Stanbic Bank, 8% fixed deposit and no mortgage rate at the time of study.

### Estimated time of service abroad

Many health professionals who leave the country do so within 5 years of graduation. It was therefore estimated that the majority, if they do not return, would spend about 30 years of working life in the recipient country.

## Results

The results are presented in sequence below starting with cost of primary education, secondary and later tertiary education. The lost investments at varying bank interest rates are shown.

### Per capita primary school costs

The cost of primary school was determined by obtaining current school fees for students at two non-governmental schools, a mission school and a private school. Each of the schools was charging MK15,000 per term (3 school terms in a year). At the prevailing foreign currency exchange rate of 1 US$ to MK 137, the total tuition per student for the whole 8 years would be = 8 years × 3 terms × MK 15,000 = MK 360,000 i.e. US$ 2627.73. This method of estimating primary education costs has been used before by Kirigia et al [10].

### Secondary school training

The cost of secondary school was estimated from averaging the cost of two schools, one private (Kaphuka Secondary School) and the other mission (Malamulo Secondary School). These schools were chosen as they were financially self-reliant, unlike government secondary schools which were heavily subsidized. Secondary school education in Malawi is for 4 years. The total tuition at secondary school would sum to 4 years × 3 terms × MK 25,000 = MK 300,000 i.e. US$ 2189.78.

### Training of enrolled nurse-midwife

As stated elsewhere in this paper, the training of enrolled nurse-midwives takes place either at mission nursing schools or the Malawi College of Health Sciences and runs for three years. Tuition and boarding facility costs at all the facilities are currently estimated at MK 206,049 each year. From about 2001, all enrolled nurse-midwife direct training costs in Malawi are paid for through finances obtained from the World Bank's Highly Indebted Poor Country program (HIPC). Students are not required to pay any tuition or boarding fees as this money is paid for by the government to the institution. The amount of money that has been agreed between government and the training institutions is MK 206, 049 annually per student. For the three years training, this would total MK 206,049 per year × 3 years = MK 61,8147.00 i.e. US$ 4512.02.

By just adding the total fees of primary, secondary and tertiary education, the total fees of training an enrolled nurse-midwife from primary to professional school would total; US$ (2627.73 + 2189.78 + 4512.02) = US$9329.53.

### Training of a registered nurse-midwife

The Kamuzu College of Nursing of the University of Malawi provides nursing degrees categorized as generic (four year degrees offered to students enrolled straight from secondary school) and post-basic (degrees offered to enrolled nurses who have at least two years of service). Nurses with three year college diplomas in nursing and one year midwifery are also enrolled in degree programs to train for an extra two years before being awarded degrees. Other offerings at the KCN that have begun in the past two years are: Bachelor of Science in Advanced Midwifery and Diploma in Nursing for enrolled nurses. For the purposes of this paper, only the cost of the generic nurse-midwife degree program will be presented and discussed.

The fees for each KCN student is about US$ 5300–US$ 5500 each year. According to Namate in 1995 [4], the fees of producing a degree nurse (without an extra year of midwifery education) at the KCN was reported to be US$ 21, 527 in 1992/93 and US$ 23,080 in 1993/94. This amount has essentially not changed much. For the purpose of this paper we will take the lower estimate i.e. US$ 21, 527, which translates to US$ 5381.75 per year. A one-year midwifery course that virtually all nurses take will add the an additional year to the basic nursing degree to reach US$ 26,908.75 for five years.

The total cost of education from primary to tertiary degree level nurse-midwife will add an additional year up to US$ 31,726.26 i.e. US$ (2627.73 + 2189.78 + 26,909.75).

### Lost investment due to migration

If the investment is assumed to be made at the time of migration, the losses of investment from the health professional at different bank interest rates can be obtained by using the following formula:

FV = Sum * (1+r)^n ^where; Sum = principal amount invested; r = compound interest rate per annum; n = the number of years the money is invested [12-14].

The results are presented in Table 1 in which the total cost of training a nurse-midwife (both enrolled and degree nurse-midwife) are presented. In Table 2, the lost investment is presented at different interest rates for both enrolled and degree nurse-midwives.

**Table 1:** Cost of Training for Nurse-Midwives in Malawi

**Stage of Training**	**Amount in US$**
Primary education	2627.23
Secondary Education	2129.78
Enrolled Nurse-Midwife Tertiary Education	4512.02
Degree Nurse-Midwife Tertiary Education	26,908.75

Total Cost for Enrolled Nurse Midwife	9329.53
Total Cost for Degree Nurse-Midwife	31,726.26

**Table 2:** Per capita lost returns from the training of nurse-midwives in Malawi

**Professional category**	**Principal Sum (US$)**	**Interest Rate in% per annum**	**Expressed as % of lost investment at 7%**
Enrolled-nurse midwife	9329.53	7	1
	9329.53	8	132
	9329.53	10	229
	9329.53	25	10,612

Degree Nurse-Midwife	31726.26	7	1
	31726.26	8	132
	31726.26	10	229
	31726.26	25	10,612

### Sensitivity analyses

From Table 2, the lost investment in training of an enrolled nurse-midwife at an investment of US$9329.53 at 7% interest rate per annum for 30 years totals US$ 71,018.76. At the same interest rate and duration of investment, the lost investment for a degree nurse-midwife amounts to US$ 241,508.38. These amounts however rise to 132% at 8% interest, 229% at 10% interest and 10,612% at 25% interest rate. The lost investment for a degree nurse-midwife is 3.4 times (340%) that of an enrolled nurse-midwife.

### Societal losses

Migration results in many other losses other than just financial. The health professionals that are left behind have a much higher workload, may deliver lower quality care as they can not spend adequate time on patients resulting in burn out. Tasks and roles that were previously identified for performance by highly skilled staff may be delegated to low cadres [15].

## Discussion

There is increasing concern as to whether the investment in education of health professionals in a country eventually helps the society that is making the investment through provision of health services [16]. We found the cost of training an enrolled nurse-midwife from primary school through nurse-midwifery training in Malawi is US$ 9,329.45. For a degree nurse-midwife, the total cost is at US$ 31,726.26. For each enrolled nurse-midwife that migrates out of Malawi, the country loses a total investment return of between US$ 71,081.76 and US$ 7.5 million at bank interest rates of 7% and 25% per annum for 30 years. For a degree nurse-midwife, the lost investment return ranges from US$ 241,508.38 to US$ 25.6 million at 7% and 25% interest rate per annum for 30 years.

The amount of lost investment depends on how high the interest rate is, the duration in lost years of service, and the principal amount invested. The amount of lost investment for nurse who emigrate from Malawi is much high than that reported by Kirigia et al [12]. While the cost of training a nurse in Kenya from primary through to nursing school was US$ 43,180, which is higher than that in Malawi, the highest interest rate in Kenya was 15.64% while in Malawi was 25%. The fact different countries may have different interest rates and even within a single country, bank rates may differ and be unstable means comparing lost investments with much precision problematic.

Developing nations health care systems are losing millions of dollars for losses in total health professional who migrate to other countries, resulting in a sort of reverse financial aid [17]. The estimated costs of migration depend to some extent on the assumptions made. If the mortgage rate (a number which is usually higher than the fixed deposit interest rates is used) the amount lost is usually higher. This is mostly because banks charge more interest against people who borrow from them but pay out much lower interest rates on fixed deposits.

Lately, there have been discussions to reimburse developing nations of lost investment in the training of health professionals. If this was considered seriously as a viable alternative to the losses in investment, there will be need to estimate what developing nations have lost through the out-migration of their health professionals. Our study indicates that the amount of lost investment would vary depending on the assumptions made and the interests rates used.

The study had several limitations. We did not discuss the return value to the country of remittances. Aside from the fact that these numbers are not officially reported, we have a concern that they are channeled only to private parties related to the migrating professionals. They are not sent to compensate a public that was taxed to support the subsidies for training schools, or to a government that could reinvest in training additional health workers with the funds. Buchan et al [18] and van Dalen et al [19] have shown that many African health professionals in Diaspora send back remittances to their families, although this is mostly through unofficial means.

Another limitation of this study is that it is not possible to quantify personal or social benefits from education for the individual, family and home society. While this study was concerned mainly with the investment losses from migration, it is instructive also to consider that primary and secondary education do benefit the individual who is education, the family and their society. For instance it has reported that a woman's advanced education is positively associated with use of modern contraception for fertility control [20,21]. This certainly has benefits at the individual, family and societal level.

The costs of education estimated in this study also exclude the capital investment costs of buildings and other equipment, and training of teachers that are necessary for education.

The calculations assume the health professionals would spend 30 years working in a recipient nation. This may not be the case as one's working life can be terminate due to a diversity of reasons.

The estimated costs of training health professionals also took into account incremental costs only. There was no factoring in of investment in the training of lecturers and tutor, infrastructural and other capital investments.

While Malawian nurses migrate out to mostly developed nations, health professionals also move into Malawi. Muula has documented that, for medical doctors, the majority of specialists practicing in Malawi in 2003 were non-nationals [22].

## Conclusion

The lost investment in training of health professionals like nurse-midwives depends on several factors which include number of years the health professional work in a particular country, the interest rates used and the actual amount of educational costs. African countries are failing to benefit from the investment in the training of their health professionals due to migration resulting in net losses of human resources for health. There is need to quantify the amount of remittances that developing nations get in return from those who migrate.

## Abbreviations

FV: Future value

KCN: Kamuzu College of Health Sciences

OECD: Organisation for Economic Co-operation and Development

SDA: Seventh-Day Adventist

## Competing interests

The author(s) declare that they have no competing interests.

## Authors' contributions

ASM conceived the study, designed study instruments, collected data, participated in analysis and drafting of manuscript. BP participated in design of data collection instrument, collected data and contributed to drafting of manuscript. FCM collected data, participated in analysis and contributed to manuscript draft. All authors have read and approved the final manuscript.

## Pre-publication history

The pre-publication history for this paper can be accessed here:


